# How Deep Is Your SNARC? Interactions Between Numerical Magnitude, Response Hands, and Reachability in Peripersonal Space

**DOI:** 10.3389/fpsyg.2018.00622

**Published:** 2018-05-01

**Authors:** Johannes Lohmann, Philipp A. Schroeder, Hans-Christoph Nuerk, Christian Plewnia, Martin V. Butz

**Affiliations:** ^1^Cognitive Modeling, Department of Computer Science, University of Tübingen, Tübingen, Germany; ^2^Department of Psychiatry and Psychotherapy, Neurophysiology and Interventional Neuropsychiatry, University of Tübingen, Tübingen, Germany; ^3^Department of Psychology, University of Tübingen, Tübingen, Germany; ^4^Leibniz-Institut für Wissensmedien (IWM), Tübingen, Germany; ^5^LEAD Graduate School, University of Tübingen, Tübingen, Germany; ^6^Werner Reichardt Centre for Integrative Neuroscience, Tübingen, Germany

**Keywords:** SNARC effect, theory of magnitude, embodied numerical cognition, virtual reality, motion capture

## Abstract

Spatial, physical, and semantic magnitude dimensions can influence action decisions in human cognitive processing and interact with each other. For example, in the spatial-numerical associations of response code (SNARC) effect, semantic numerical magnitude facilitates left-hand or right-hand responding dependent on the small or large magnitude of number symbols. SNARC-like interactions of numerical magnitudes with the radial spatial dimension (depth) were postulated from early on. Usually, the SNARC effect in any direction is investigated using fronto-parallel computer monitors for presentation of stimuli. In such 2D setups, however, the metaphorical and literal interpretation of the radial depth axis with seemingly close/far stimuli or responses are not distinct. Hence, it is difficult to draw clear conclusions with respect to the contribution of different spatial mappings to the SNARC effect. In order to disentangle the different mappings in a natural way, we studied parametrical interactions between semantic numerical magnitude, horizontal directional responses, and perceptual distance by means of stereoscopic depth in an immersive virtual reality (VR). Two VR experiments show horizontal SNARC effects across all spatial displacements in traditional latency measures and kinematic response parameters. No indications of a SNARC effect along the depth axis, as it would be predicted by a direct mapping account, were observed, but the results show a non-linear relationship between horizontal SNARC slopes and physical distance. Steepest SNARC slopes were observed for digits presented close to the hands. We conclude that spatial-numerical processing is susceptible to effector-based processes but relatively resilient to task-irrelevant variations of radial-spatial magnitudes.

## Introduction

Relational inference is fundamental for adaptive behavior control. Catching a flying object requires an estimate of the hand position in space and time as well as the velocity of the object. Even simple grasping movements require a thorough estimate of the distance between the target object and the own body. From a conceptual point of view, these estimates are similar since they all require magnitude judgments – in time, space, and the respective derivatives thereof, that is, speed and acceleration. There is indeed evidence for a common metric involved in the representation of time, space, and quantity. According to *a theory of magnitude* (ATOM; Walsh, 2003), this metric evolves from the sensorimotor system and resides primarily in the parietal cortices of the brain (Walsh, 2003; Bueti and Walsh, 2009; Cohen-Kadosh and Dowker, 2015). According to ATOM, the common magnitude metric emerges in the service of action control and develops into a general magnitude system that can be used to represent arbitrary quantities, for instance, in terms of numbers. Hence, ATOM can account for the apparent overlap of magnitude processing across modalities.

One example for such an overlap with respect to space and magnitude is the well-studied spatial-numerical associations of response codes (SNARC) effect. The SNARC effect shows a strong interaction between directional spatial information in the left-hand/right-hand of responding and the numerical magnitude information as semantically displayed in numerical symbols (Dehaene et al., 1993; Wood et al., 2008). In simple judgment tasks on numerical magnitude or parity, small numbers are faster responded to with the left hand than with the right hand, and vice versa for large numbers. SNARC effects can be obtained with different response systems such as hands, eyes, or feet (Fischer et al., 2003; Schwarz and Müller, 2006; Hesse and Bremmer, 2016), for different modalities and number notations (Nuerk et al., 2005), and SNARC effects can also influence overt action decisions, which nicely demonstrates the relevance of the metrical overlap for action coordination in more or less naturalistic settings (Shaki and Fischer, 2014; Schroeder and Pfister, 2015). Furthermore, interactions have been documented between the spatial information triggered by different magnitudes such as auditory and visual intensity (Fairhurst and Deroy, 2017) or by number and musical pitch in both factorial designs (Weis et al., 2016) as well as in dual-task situations (Fischer et al., 2013). These findings are consistent with the assumption of a common magnitude representation, which is assumed to be located within the horizontal segment of the intraparietal sulcus (Dehaene et al., 2003), and highlight the overlap between numerical and spatial cognition.

Usually, the SNARC effect has been interpreted in terms of a mental number line, oriented horizontally, with small numbers represented to the left of large numbers. However, different studies have shown that spatial associations of numerical magnitude are not restricted to the horizontal dimension, but can be extended to the vertical and radial dimension as well. ATOM can account for the existence of all of these mappings, by stating that magnitudes are flexibly mapped on spatial dimensions involved in the task. According to this line of reasoning, for instance radial SNARC effects can arise because nearby space corresponds with a small movement amplitude and thus shares the meaning of small magnitudes with small numbers. This implies that spatial-numerical mappings are more flexible and are not restricted to a single, horizontal representation. However, ATOM does not provide a prediction, which kind of mapping is applied under which circumstances. From an anticipatory behavior control perspective (e.g., Hoffmann, 1993, 2003), the application of a certain mapping should not occur automatically, but should be driven by task relevance. Accordingly, we pursued two broad aims with the current study. First, we wanted to corroborate further evidence for a sensorimotor grounding of SNARC effects. Second, we wanted to investigate the situatedness of spatial-numerical mappings in task-relevant and task-irrelevant spatial dimensions. In order to do so, an experimental setup would be desirable that allows to contrast different spatial axes within the same environment, and which provides a natural user interface. Hence, we realized a SNARC setup in an immersive virtual reality (VR), combined with online motion capture.

### How Deep Is the SNARC Effect?

Already the first scientific description of SNARC-like effects included rather diverse (and partially complicated) introspective self-reports of mental number lines wandering through space, also extending to the radial depth dimension (Galton, 1880). However, to date, only relatively few studies have tested other spatial directions than the horizontal left-to-right plane, or even tested combinatory-factorial experimental designs to investigate interactions between the potentially available horizontal, vertical, or radial (distance-based or sagittal) SNARC effects (for an exhaustive review, see Winter et al., 2015). When studied in isolation, spatial-numerical associations were observed (at least in Western cultures and besides the left-to-right direction) for lower-hand vs. upper-hand (but not feet) responses from bottom-to-top (Hartmann et al., 2012; Wiemers et al., 2017) and also when responses were mapped from back-to-front (i.e., vertical in the sense of close/far from the body; Ito and Hatta, 2004; Shaki and Fischer, 2012). However, in traditional setups using fronto-parallel two-dimensional computer monitors for presentation of stimuli, the metaphorical and literal interpretation of close/far (along with the linguistic declaration thereof) are not necessarily distinct. This is problematic because also vertical labels and horizontal response arrangements can produce spatial-numerical associations (Holmes and Lourenco, 2011). Since spatial associations in different spatial dimensions could have different cognitive origins (Winter et al., 2015; Wiemers et al., 2017), it is not clear whether which dimensions would produce an effect or how the different spatial and numerical magnitudes would interact. Nevertheless, at least semantically, there seems to be an association between close-small and far-large (Santens and Gevers, 2008). Results implying the presence of radial SNARC effects circulating the body have been reported by Marghetis and Youngstrom (2014). In their study, participants had to judge the magnitude of single-digit numbers by stepping forward or backward. Marghetis and Youngstrom (2014) compared performance in the magnitude judgment for whole numbers (1 to 9, except 5) and integers (-9 to 9, without 0). In the latter task, a SNARC-like pattern emerged, with backward responses being faster for negative numbers, and forward responses being faster for positive numbers. However, if the stimulus-set only contained positive numbers, no association between magnitude and movement direction was observed.

As it was pointed out by Winter et al. (2015), the multitude of flexible spatial-numerical mappings and their task-dependency renders numerical cognition highly situated. Furthermore, the reviewed findings imply that numerical cognition builds upon a rich spatial representation, which can also exceed implicit directional SNARC effects on other, explicit linkages and in effects of spatial extension (Patro et al., 2014; Cipora et al., 2015). According to ATOM, this spatial representation is the same that is used for behavior control. Indeed, there is some evidence for a close relation between the multisensory spatial mappings used to represent the space surrounding the body – the so-called peripersonal space (e.g., Holmes and Spence, 2004) – and numerical space. Longo and Lourenco (2010) investigated whether biases of lateralized attention within peripersonal space also apply to numerical cognition. In pen-and-paper line bisection, a small leftward bias is typically observed for lines close to the body, which reverse to a rightward bias with increasing distance. Longo and Lourenco (2010) observed the same bias and a similar effect of physical distance if participants had to bisect number pairs. Furthermore, the size of both biases was highly correlated on an individual level. This implies a close coupling of the representation of physical and numerical space. Further evidence for this coupling was provided by Patro et al. (2015), who showed that counting directions in preliterate children are emphasized in peripersonal space. Moreover, it could be that the flexible change between different egocentric and allocentric perspectives and the transformations between peripersonal and extrapersonal spaces contribute to the effects of embodied numerical learning paradigms (Dackermann et al., 2017). Together, these findings imply a highly flexible representation, which is used to map numbers and space, and that this representation is closely tied to the representation of physical space, which is grounded in sensorimotor experience.

Further evidence for the sensorimotor gounding of numerical representations proposed by ATOM comes from studies implying SNARC-like number-action links. For instance, it has been shown that numerical magnitude can afford compatible grip apertures (Andres et al., 2004). In this study, participants had to close or open their hand in response to a digit’s parity. Closure was faster in case of small digits, while opening was faster in case of large digits. In a similar vein, it has been shown that large digits afford power grasps, while small digits afford precision grasps – even if the numerical magnitude is not task relevant (Lindemann et al., 2007).

Regarding *interactions* between SNARC effects with different spatial codes in the response dimension, only few studies have previously pitted different spatial dimensions against each other, and if they did so, diagonal response mappings were used (Gevers et al., 2006; Holmes and Lourenco, 2011, 2012). Noteworthy, the perceptual presentation of semantic magnitudes (in form of Arabic single-digits) was mostly carried out using two-dimensional stimuli on flat computer displays, varying only the spatial response dimension in horizontal, vertical, or radial direction. However, regarding SNARC effects with different spatial codes in terms of visual-perceptual presentation, to the best of our knowledge, there was no systematic investigation up to now. In the present study, we investigated whether the possibility of concurrent extensions on the two-dimensional fronto-parallel and three-dimensional proximal-distal plane yields a more complicated and possibly interacting scheme of a single-digit’s spatial associations.

From the available literature, two main hypotheses can be formulated. If associations between spatial and numerical magnitudes are driven by direct mappings of perceptual magnitudes on spatial directions, there should be crossmodal interactions at the level of the theoretical core magnitude system, as it was also repeatedly found for other magnitude dimensions (Fischer et al., 2013; Weis et al., 2016; Fairhurst and Deroy, 2017) or for the direct comparison between semantic magnitude and physical extension in the size congruity effect (Henik and Tzelgov, 1982). The interactions should be detectable even if different psychophysical scales for the distinct spatial dimensions might result in different magnitude weights (see Winter et al., 2015 for a similar argument). However, considering the previous results implying a relation between physical and numerical space (Longo and Lourenco, 2010; Patro et al., 2015; Dackermann et al., 2017), changes in reachability or the transition from peri- to extra-personal space might result in a more complex modulation of SNARC effects along the radial axis.

### Embedding Numerical Cognition in Virtual Reality: The Present Study

In the present study, we introduce a VR scenario to systematically investigate the interaction between perceptual distance and horizontal SNARC effects. Compared to classic, fronto-parallel display setups used to study SNARC effects, VR allows to vary perceptual distance without confusion with the vertical dimension in a three-dimensional stereoscopic simulation. This allows the combination of a horizontal response mapping with stimulus presentation on the radial axis and hence, spatial codes in the response and presentation dimensions can be varied experimentally. Furthermore, the incorporation of online motion capture allows the implementation of a natural, continuous response mode, as well as sensorimotor exploration of the task space.

Two distinct procedures were carried out in the present research. First, although the simulation in VR already includes stereoscopic 3D images (using the Oculus Rift^©^ DK2 head-mounted display), we furthermore included a sensorimotor exploration phase prior to the actual SNARC experiment to provide an immediate experience of peripersonal space in the VR test environment, and possibly adjust for individual differences in overestimation of perceived reachability (Fischer, 2005). To that end, in our implementation, the Leap Motion^©^ near-infrared sensor was used to track and stream hand movements to the VR scenario. Such setups, which allow participants to explore the VR with a body representation, have previously shown to increase the degree of immersion and spatial perception within the VR (Mohler et al., 2008; Linkenauger et al., 2015). Furthermore, there is evidence that the distinction between peri- and extra-personal space remains valid in suitable VR setups (Gamberini et al., 2008). Second, in order to obtain an action-related, kinematic measure of the response activation during the task, and closely following the results of contralateral motor activation in the incongruent conditions of the SNARC effect (e.g., Keus et al., 2005), we used a slightly different effector response than in previous studies, which had mostly utilized response box key presses. More precisely, the response mode in the current experiment was realized by asking participants to close their hands, which were positioned at a fixed and comfortable distance in the VR display. Thus, this response modality further allowed for continuous response activation in conflicting conditions, next to the established assessment of SNARC effects by means of response times (RTs) and regression coefficient analysis. In a comparable VR setup using the same equipment, we were previously able to reproduce the behavioral bias for food stimuli (Schroeder et al., 2016).

To conclude the motivation for the current study, the concurrent assessment of SNARC effects in the three-dimensional VR environment – including spatial displacements within and outside reachable space – allowed us to investigate interactions between spatial-numerical mappings on radial and horizontal axes. A direct mapping approach would predict a linear relationship between numerical magnitude and spatial magnitude on the radial axis. Precisely, in this case, left-side responding should be faster for small semantic digits (horizontal SNARC), but also for digits appearing closer to the participants (radial SNARC), and vice versa for right-hand responding. If SNARC effects are tied to spatial representations used in behavior control, as proposed by ATOM, a non-linear relation between numerical magnitude and radial distance – indicating effects of reachability or the transition from peri- to extra-personal space – seems more likely. In order to investigate these two hypotheses, we had participants perform a magnitude judgment with respect to digits appearing at different distances on the radial axis within or outside peripersonal space. In a first experiment, we analyzed the interactions between the SNARC effect and physical distance by applying 10 equidistant spatial displacements. In a second experiment, we focused on the four most relevant displacements identified in the first study.

## Materials and Methods

### Participants

Sixteen students from the University of Tübingen participated in the first experiment (seven females). Their age ranged from 19 to 30 years (*M =* 22.3, *SD* = 2.8). All participants were right-handed and had normal or corrected-to-normal vision. Participants provided informed consent and received either course credit or a monetary compensation for their participation. For the second experiment, another 16 participants were recruited (10 females), none of whom participated in the first experiment. Their age ranged from 19 to 29 years (*M* = 22.0, *SD* = 2.8). Again, all participants were right-handed and had normal or corrected-to-normal vision. They provided informed consent and were compensated with course credit or money for their participation. Both experiments were conducted in accordance with the Code of Ethics of the World Medical Association (Declaration of Helsinki).

### Apparatus

To immerse participants in the VR, they were equipped with an Oculus Rift^©^ DK2 stereoscopic head mounted display (HMD; Oculus VR LLC, Menlo Park, CA, United States). Motion tracking of hand movements was realized with a LeapMotion^©^ near-infrared sensor (LeapMotion, Inc., San Francisco, CA, United States; SDK version 3.1.3). The LeapMotion^©^ sensor provides positional information regarding the palm, wrist, and phalanges. This data can be used to render a hand model in VR. Furthermore, the API provides a measure of the hand closure of the respective hand, ranging between zero (open hand) and one (clenched fist). This measure of hand closure was used to determine the response in the SNARC task. A response was collected if the respective value was larger than 0.75. The whole experiment was implemented within the Unity^®^ engine 5.5.0 using the C# interface provided by the API. To allow the experimenter to observe the scene and to assist the participants, the VR scene was rendered in parallel on the Oculus Rift and a computer screen.

### Virtual Reality Setup

**FIGURE 1:**
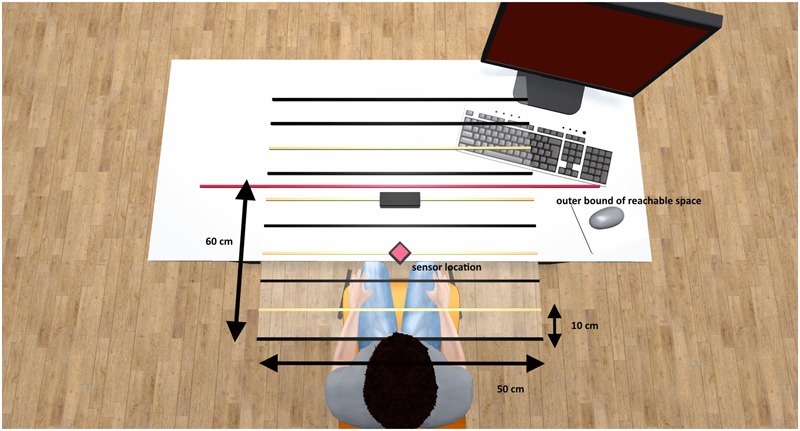
Physical setup and extent of the task space. The red diamond indicates the sensor position and the red line indicates the outer bound of the reachable space. The lines correspond to the distance indicators in the VR environment. Yellow lines indicate the four distances that were applied for stimulus presentation in both experiments. Spacing between adjacent lines was about 10 cm.

The VR setup put participants on a meadow surrounded by hills and various trees. A black plane covered with equally spaced white lines appeared in front of them. These lines indicated the displacements in the radial plane where the stimuli for the exploration and the magnitude judgment would appear. We chose these discrete distance indicators to make the distinction between reachable and not-reachable space even more salient. The distance between adjacent lines was about 10 cm. The center of the tracking range of the LeapMotion^©^ sensor corresponded with the fourth line in the setup (second experiment: second line). The outer, radial limit of the tracking range was indicated by a cardboard box to provide participants with haptic feedback regarding the bounds of the task space (calibrated with the sixth/third visual horizontal line in the VR and with the tip of the middle finger with maximally extended arm). Please note that the interaction range was limited by the sensor range, which covered about 60 cm in depth and 50 cm from left to right, and not by the length of participants’ arms. The real-world setup of the task space is shown in **Figure 1**. Instructions and feedback were presented on different text-fields, aligned at eye-height.

### Procedure

**FIGURE 2:**
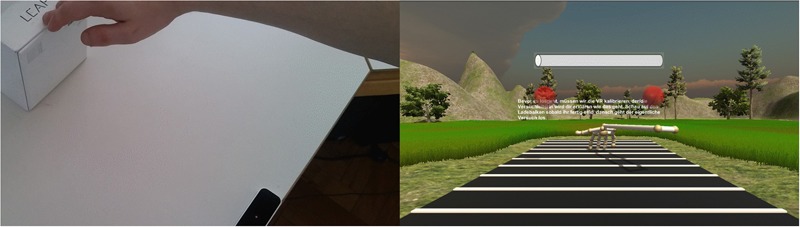
The calibration setup. The virtual environment is shown in the right panel and the white lines correspond to the lines shown in **Figure 1**. At the beginning of the experiment, the height of the sensor origin was adjusted in such a way that a stretched arm (left panel) yielded the impression shown in the right panel. As it is shown in the right panel, four displacements were outside the reachable space. The red spheres indicate the initial hand positions for the magnitude judgment, in the calibration phase it was assured that these positions could be reached and held conveniently.

At the beginning of the experiment, participants received a verbal instruction regarding the VR equipment. Then, the HMD was put on and the experiment started. In a first step, the scene was calibrated according to the participant’s height and arm length, that is: the ground position was adjusted in a way that the hand appeared above the task space when it was stretched out to the outer bound of the reachable space (see **Figure 2**). In order to do so, participants had to stretch out their dominant right arm and place the tip of their middle finger on the top of a card box, which was placed at the border of the LeapMotion^©^ sensor’s tracking range. If necessary, the experimenter gently corrected the participant’s seated position to assure that his or her arm were maximally extended to reach the box (see **Figure 2**, left panel). Next, the experimenter adjusted the visual position of the virtual hand model to assure that the virtual hand appeared above the task space (see **Figure 2**, right panel). Furthermore, it was assured that the response hand positions for the SNARC task could be reached conveniently. This procedure ensured that participants experienced a standardized reachability limit in VR and the calibration furthermore reduced the influence of reaching range overestimations (Fischer, 2005).

The experiment consisted of two parts. First, participants performed an exploration task. This was intended to familiarize the participants with the sensorimotor mapping and to provide an experience of reachability. Second, participants performed two blocks of a magnitude judgment task within the VR. Participants could practice the magnitude judgment for 20 trials before the actual blocks started. Both tasks are described in detail below. After the experiment, participants were asked to complete a presence questionnaire (IPQ; Schubert et al., 2001). The whole procedure took 90 to 120 min, including preparation and practice trials.

#### Sensorimotor Exploration Phase

To familiarize the participants with the sensorimotor mapping with respect to the different displacements and to enhance their depth perception, participants performed a reaching task within the VR. In this task (presented in the same environment as the later magnitude judgment task), colored spheres appeared at different spatial displacements, indicated by horizontal lines. Participants had to touch the spheres with the fingertips of their left or right hand. The color of the spheres indicated the requested hand: participants had to touch yellow spheres with their left hand and green spheres with their right hand. Upon touching, the spheres emitted a flashing burst. If participants touched the sphere with the correct hand, the flash was white. If they touched the sphere with the wrong hand, the flash was red.

**FIGURE 3:**
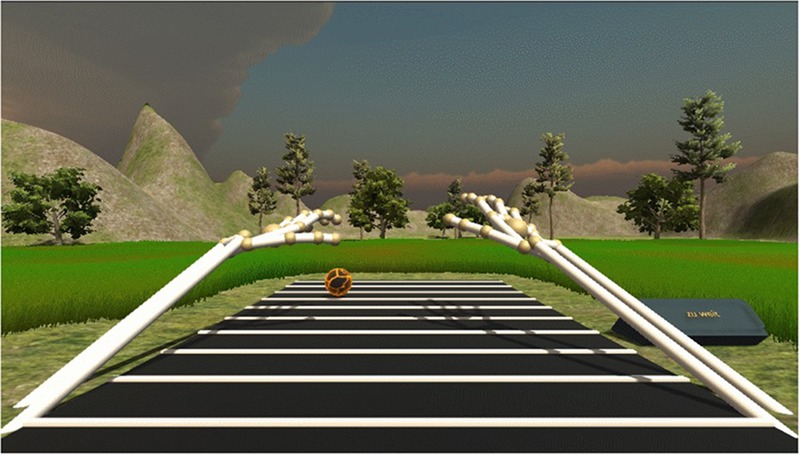
The sensorimotor exploration task. Colored spheres appeared at different displacements and participants were requested to touch them with the correct hand (yellow spheres = left hand, green spheres = right hand). If a sphere appeared at an unreachable distance, participants were requested to push the button on the right side, labeled as “too far” (“zu weit,” in German). This task was intended to familiarize the participants with the VR environment and to provide a behavioral experience of reachability and distance.

If the spheres appeared at unreachable distances (displacements 7–10 in the first experiment, displacement 4 in the second experiment), participants were requested to press an accordingly labeled button (“too far,” German: “zu weit”) on the right side of the task space. The setup for the sensorimotor exploration task is shown in **Figure 3**. Participants had to perform 10 reaching movements per displacement, five with the left and five with the right hand, yielding 100 trials in the first experiment (10 different displacements) and 40 trials in the second experiment (four different displacements). The 10 repetitions per distance sampled the whole width of the task space, covering the left and right space. Participants had to perform ipsilateral as well as contralateral reaching movements. The order of presentation was randomized and error trials were not repeated. The performance in this task was not evaluated, because the exploration was only intended to familiarize the participants with the environment and to provide a behavioral experience of reachability and distance.

#### Magnitude Judgment Task

After the sensorimotor exploration phase, participants were requested to perform two blocks of a magnitude judgment task. Here, they had to repeatedly classify single-digits (1–4, 6–9) as being either smaller or larger than 5 by clenching their left or right fist. The response mapping varied between the two blocks: in one block, participants had to clench their right fist in case of digits larger than 5 and their left fist in case of digits smaller than 5. This mapping was reversed in the other block. The order of the response mapping was randomized.

**FIGURE 4:**
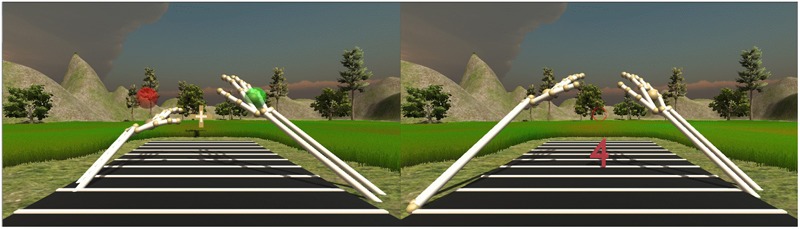
The magnitude judgment task. Preconditions **(left**): open palms had to be placed correctly into initial positions (spheres turning green) and head rotation had to focus the outer-bound fixation cross for 2000 ms. Trial **(right**): a single-digit target at one of the displacements had to be classified as being smaller or larger than 5. Participants had to respond by clenching their fist as fast as possible while keeping their hands at the initial positions.

Both blocks in both experiments consisted of 320 trials and each trial consisted of two parts. At the beginning of a trial, participants had to move their hands into initial positions, indicated by red, semi-transparent spheres, and located at the fourth (first experiment), or the second displacement (second experiment), respectively (see **Figures 2, 4**). If the palms were within the positions and the respective hands were open, the spheres turned green. Furthermore, participants had to center their field of view on a fixation cross located at the outer bound of the task space. The inner part of the fixation cross turned green once the center of the visual field had been directed toward the fixation cross for at least 2000 ms (see **Figure 4**, left panel). When these preconditions were met, the spheres and the fixation cross disappeared and after a SOA of 250 ms the target digit appeared at the center of one of the 10 (first experiment) or four (second experiment) displacement indicators (see **Figure 4**, right panel). Red, 3D mesh models of Arabic single-digits (1–9, except 5) were used as target stimuli. Digits were 7.7 cm in height and subtended a visual angle of 19.5°, 15.20°, 12.45°, 10.55°, 9.15°, 8.08°, 7.23°, 6.54°, 5.97°, and 5.50° at the different presentation distances, respectively.

Trials were canceled if the response took longer than 2000 ms. Furthermore, trials were canceled if the hands left the initial position or if either hand was clenched during the 250 ms SOA between the offset of the fixation cross and the presentation of the target stimulus (0.35%/0.38% of all trials in the first experiment/second experiment). The respective trials were repeated at the end of the block. In case of time-outs (more than 2000 ms), early movements (less than 250 ms, that is, within the SOA), or wrong responses, participants received according feedback. If the response was correct, participants received positive feedback. The whole experiment was self-paced, since trials only started when participants took the initial position and fixated the fixation cross. Hence, participants could (and they were encouraged to) take breaks between trials at any time, but they were not allowed to take off the HMD during breaks. All participants tolerated the VR procedure well and no experimental session was canceled.

Participants could practice the magnitude judgment before the actual blocks. In these training trials, participants responded with their left hand in case of a small (1) and with their right hand in case of a large practice digit (10); note that the large practice digit was not part of the actual stimulus set during testing. After completing 20 trials correctly, participants were allowed to proceed with the actual blocks.

### Factors, Measures, Data Treatment

In both experiments, we varied two factors across trials and one factor across blocks. First, the *spatial displacement* of the target digit in the radial axis varied. In the first experiment, 10 equally-spaced radial displacements were used. The physical distance between two adjacent displacement indicators was about 10 cm (see **Figure 1**). In the second experiment, only four out of the 10 initial displacements were used; here, the physical distance between two adjacent distance indicators was about 20 cm (yellow lines in **Figure 1**). Second, the *digit magnitude* varied, we used the digits from 4 to 4 and 6 to 9 as target stimuli. Third, the response mapping varied between blocks, in one block participants responded with the left/right hand to small/large stimuli, in the other block, this mapping was reversed. In the analysis, this factor was recoded as *response hand* – either left or right. Each of the 80 (first experiment) or 32 (second experiment) displacement × digit combinations were repeated 4 (first experiment) or 10 (second experiment) times per block, yielding 320 trials per block. Trial and block order was randomized. We recorded correct response times (RTs) in the magnitude judgment task and computed medians for all factor combinations. Furthermore, we recorded the maximum hand closure (MHC) of the irrelevant (incorrect) hand in each trial, as well as the respective time of the maximum hand closure time (MHCT). The MHC measure was thought to roughly reflect the degree of involuntary response preparation amid eventually correct responding especially for incongruent trials. Data from error trials were excluded from the analyses (4.2% in the first experiment and 4.7% in the second experiment). Before the analysis, RT outliers above or below two standard deviations from the respective cell mean were excluded as well (0.2% in the first experiment^1^ and 3.8% in the second experiment).

## Results

Seeing that the first and second experiment only differed regarding the number of spatial displacements, to focus the analysis, and to increase the statistical power, we here report the results from the combined analysis with the between factor *experiment* for all *N* = 32 participants, considering only the four displacements applied in both experiments (close to the body, close to the hands, at the border between peripersonal and extrapersonal space, and in extrapersonal space). To anticipate, the between-experiments factor was not significant in any analysis and results were overall comparable. We report repeated measures ANOVAs and regression coefficient analyses based on RTs, MHC, and MHCT data. All ANOVAs were carried out with type III Sums of Squares. In case of violations of the assumption of sphericity, the respective *p*-values were submitted to a Greenhouse–Geisser correction. All *p*-values obtained from *post hoc t*-tests were submitted to a Bonferroni–Holm adjustment to correct for multiple comparisons. Data from the IPQ questionnaires was compared with reference data using independent sample *t*-tests regarding the three scales spatial presence, involvement, and realism.

### Response Times

The repeated-measures ANOVA on median RTs joined from both experiments yielded a significant main effect of *digit magnitude* [*F*(7,210) = 15.04, *p* < 0.001, $\eta_{p}^{2}$ = 0.33]. The two-way interaction between *digit magnitude* × *response hand* [*F*(7,210) = 23.17, *p* < 0.001, $\eta_{p}^{2}$ = 0.44] was significant as well. Further inspection of the main effect of *digit magnitude* revealed a numerical distance effect in terms of slower responses to digits 4 (606 ms) and 6 (620 ms), respectively, compared to 1 [564 ms; *t*(31) = 6.65, *p* < 0.001] and to 9 [569 ms; *t*(31) = 6.93, *p* < 0.001]. The two-way interaction effect between *response hand* × *digit magnitude* indicated the typical horizontal SNARC effect: judgments for relatively small digits (less than 5) were faster performed with the left hand (552 ms) than with the right hand [616 ms; *t*(31) = 6.17, *p* < 0.001]. Vice versa, responses for large digits (greater than 5) were faster for the right hand (558 ms) than for the left hand [624 ms; *t*(31) = 4.87, *p* < 0.001].

There was no indication of a radial SNARC effect in the radial viewing dimension in the two-way interaction between *spatial displacement* × *response hand* [*F*(3,90) = 0.27, *p* = 0.812, $\eta_{p}^{2}$ = 0.01]. Furthermore, the three-way interaction for *digit magnitude* × *spatial displacement* × *response hand* was not significant [*F*(21,630) = 1.18, *p* = 0.308, $\eta_{p}^{2}$ = 0.04].

In line with generally comparable data sets, the between-subjects main effect *experiment* was not significant [*F*(1,30) = 1.68, *p* = 0.205, $\eta_{p}^{2}$ = 0.05]. Importantly, both the four-way interaction between *experiment* × *spatial displacement* × *digit magnitude* × *response hand* [*F*(21,630) = 0.81, *p* = 0.609, $\eta_{p}^{2}$ = 0.03] and the three-way interaction between *experiment* × *digit magnitude* × *response hand* [*F*(7,210) = 1.08, *p* = 0.359, $\eta_{p}^{2}$ = 0.03] were not significant as well, suggesting comparable SNARC effects for the two data sets. However, there was a trending two-way interaction between *response hand* × *experiment* [*F*(1,30) = 3.10, *p* = 0.088, $\eta_{p}^{2}$ = 0.09] and participants in the first experiment were in general somewhat faster for right-hand responses (mean dRT = -9.7 ms), opposite to the behavior of participants in the second experiment (mean dRT = 8.5 ms). Finally, the ANOVA also revealed a trending main effect of *spatial displacement* [*F*(3,90) = 2.90, *p* = 0.057, $\eta_{p}^{2}$ = 0.09]: participants were fastest if target stimuli were presented at the border of peripersonal space (580 ms) as compared to the displacements close to the body [587 ms; *t*(31) = 1.83, *p* = 0.153], close to the hands [591 ms; *t*(31) = 2.30, *p* = 0.071], and compared to the presentation in extrapersonal space [591 ms; *t*(31) = 3.06, *p* = 0.027].

**FIGURE 5:**
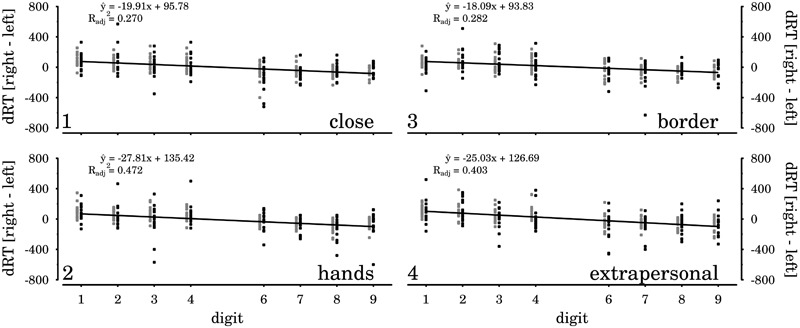
Response hand RT differences (dRT = right hand RT – left hand RT), per digit (x-axis) in the first experiment (black color) and the second experiment (gray color), for the four spatial displacements considered in the combined data analysis.

We next inspected the modulation of horizontal SNARC effects by the visual presentation of targets at the different spatial displacements. Following the standard linear regression procedure for assessing SNARC effects (Lorch and Myers, 1990; Fias et al., 1996), we separately extracted for each participant and each of the four spatial displacements the correlation coefficient between numerical magnitude and response hand RT difference (dRT = right hand RT – left hand RT). More precisely, in this regression coefficient analysis, the response hand RT differences are predicted by the numerical magnitude factor (1, 2, 3, 4, 6, 7, 8, 9). Negative coefficients are indicative of relatively faster left-hand responses to smaller digits and of relatively faster right-hand responses to larger digits, which realizes the substantial result of the horizontal SNARC effect (see **Figure 5**).

**FIGURE 6:**
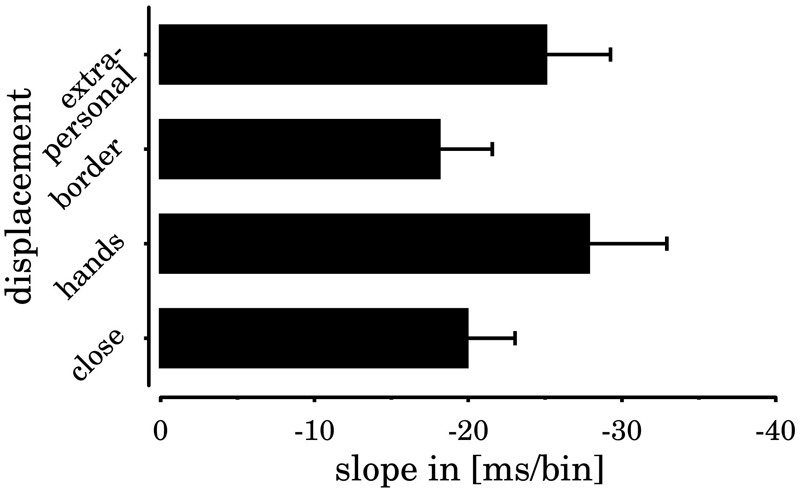
SNARC slopes for the different spatial displacement conditions. Error bars indicate the standard error of the mean. All slopes differed significantly from zero.

Throughout both studies and across all four *spatial displacements*, the regression coefficient analysis yielded negative signed coefficients, as expected for horizontal SNARC effects (means and test statistics are reported in **Table 1**, data are shown in **Figures 5, 6**). All extracted coefficients were submitted to a mixed ANOVA comprising the repeated measures factors *spatial displacement* and the group variable *experiment*. The analysis yielded a significant main effect of *spatial displacement* [*F*(3,90) = 3.60, *p* = 0.026, $\eta_{p}^{2}$ = 0.11]. The two-way interaction of *spatial displacement* × *experiment* was not significant [*F*(3,90) = 0.83, *p* = 0.481], and we neither observed a simple main effect of *experiment* [*F*(1,30) = 0.04, *p* = 0.836].

**Table 1:** Horizontal SNARC effects resulting from the regression coefficient analysis for both studies at the four considered displacements (means and standard deviations in ms/magnitude bin and in hand closure unit/magnitude bin).

	RT		MHCT		MHC	
Displacement	*M*	*SD*	*M*	*SD*	*M*	*SD*
Four extrapersonal space	-25.0^∗∗^	23.9	-19.6^∗∗^	24.8	-0.0033^∗^	0.0080
Three border	-18.1^∗∗^	19.6	-8.4^∗^	23.1	-0.0024^∗^	0.0069
Two close to hand	-27.8^∗∗^	28.9	-25.3^∗∗^	27.7	-0.0023^∗^	0.0049
One close to body	-19.9^∗∗^	17.7	-10.9^∗∗^	23.4	-0.0018	0.0060

*Asterisks indicate that coefficients differ significantly from zero. ^∗^*p* < 0.05; ^∗∗^*p* < 0.005 (one-tailed).*

In general, the results show a relatively complex modulation of horizontal SNARC effects by spatial displacement (cf. **Table 1**). Paired *t*-tests were performed to compare SNARC effects for the different displacements. The SNARC effect close to the hands was significantly larger than the SNARC effect in the border-condition [*t*(31) = -3.16, *p* = 0.012], and tended to be larger than the SNARC effect in the close-to-body condition [*t*(31) = -1.88, *p* = 0.082]. Furthermore, the border-condition SNARC effect tended to be smaller than the SNARC effect in extrapersonal space [*t*(31) = 2.14, *p* = 0.082]. All remaining comparisons were statistically not significant (*t*s < 1.55).

### Maximum Hand Closure (MHC) and Maximum Hand Closure Time (MHCT)

Based on the response-related conflict elicited by SNARC effects in different previous EEG studies (e.g., Keus et al., 2005), and previous results on number-action links (e.g., Andres et al., 2004), we expected to observe a tendency for spatial-numerical associations also in the continuous activation of responding (i.e., closing the hand) in the SNARC-*congruent*, yet *false* response (i.e., in the incongruent block as opposed to the congruent block). To inspect this potential behavior, the continuous closure of the incorrect hand during correct responding was recorded in the VR framework as dependent variable^2^. Based on this trajectory, we obtained the MHC per trial, as well as the according time (MHCT), relative to target onset.

For approximately one quarter of all participants (N _First_ = 3, N _Second_ = 4), this sort of analysis was not possible because these participants kept their incorrect hands perfectly open during responding and thus the value was continuously zero. For the remaining *N* = 25 participants^3^, data were submitted to the ANOVA and regression coefficient analysis as before, using MHC and MHCT as dependent variables.

**FIGURE 7:**
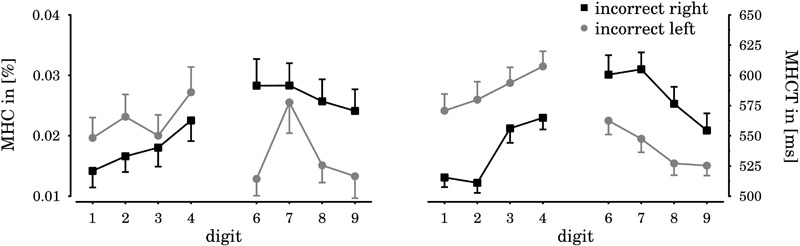
Horizontal SNARC effects for the [m]aximum [h]and [c]losure (MHC, **left**) of the SNARC-congruent, but false response, as well as the according times (MHCT, **right**). If responses had to be given with the left hand, participants clenched their right hand significantly stronger and later in case of large digits, compared to small digits (black squares). If participants had to respond with the right hand, they clenched their left hand significantly stronger and later in case of small digits, compared to large digits (gray circles). Error bars indicate the standard error of the mean.

The ANOVA on MHC revealed a significant two-way interaction between *numerical magnitude* × *response hand* [*F*(7,161) = 2.92, *p* = 0.007, $\eta_{p}^{2}$ = 0.11; see also **Figure 7**]. *Post hoc t*-tests revealed a tendency for a horizontal SNARC effect. If participants had to respond to relatively large digits (greater than 5), incorrect closure of the right hand was stronger (0.027) than incorrect closure of the left hand [0.017; *t*(24) = 2.14, *p* = 0.043]. In case of relatively small digits (less than 5), the respective difference was not significant [*t*(24) = 0.81, *p* = 0.428], but the overall pattern of results fitted a typical horizontal SNARC effect (see **Figure 7**). As before, there was no significant indication of a radial SNARC effect and the two-way interaction between *spatial displacement* × *response hand* was not statistically significant [*F*(3,69) = 1.67, *p* = 0.181, $\eta_{p}^{2}$ = 0.07]. The three-way interaction between *numerical magnitude × spatial displacement* × *response hand wa*s not statistically significant, either [*F*(21,483) = 1.43, *p* = 0.182, $\eta_{p}^{2}$ = 0.06], as well as the main effect for *numerical magnitude* [*F*(7,161) = 1.80, *p* = 0.146, $\eta_{p}^{2}$ = 0.07]. There was no main effect for the between factor experiment [*F*(1,23) = 0.87, *p* = 0.361, $\eta_{p}^{2}$ = 0.04], and no interactions involving this factor reached significance (*p*s > 0.12).

As with RTs, we also performed regression coefficient analysis and obtained consistently negative-signed coefficients (see **Table 1**). In contrast to the RT analyses, the differences between coefficients on MHC were not significant (*t*s < 0.88, *p*s > 0.39). Furthermore, *t*-tests against zero detected only a trending significance for the negative-signed regression coefficient in the condition close to the body [*t*(24) = 1.50, *p* = 0.074; one-tailed]. Horizontal SNARC effects themselves were significantly smaller than zero in all remaining conditions [close to the hands: *t*(24) = 2.36, *p* = 0.014; at border: *t*(24) = 1.72, *p* = 0.049; in extrapersonal space: *t*(24) = 2.05, *p* = 0.026].

Regarding MHCT, the ANOVA revealed significant main effects of *digit magnitude* [*F*(7,161) = 8.31, *p* < 0.001, $\eta_{p}^{2}$ = 0.27] and *spatial displacement* [*F*(3,69) = 2.77, *p* = 0.048, $\eta_{p}^{2}$ = 0.11]. The two-way interaction between *digit magnitude* × *response hand* [*F*(7,161) = 7.63, *p <* 0.001, $\eta_{p}^{2}$ = 0.25] was significant as well. All remaining effects did not reach significance (*p*s > 0.10). *Post hoc t*-tests for the main effect of *digit magnitude* revealed slower responses to digits four (586 ms) and six (581 ms), respectively, compared to one [543 ms; *t*(24) = 6.26, *p* < 0.001] and to nine [539 ms; *t*(24) = 5.51, *p* < 0.001], thus mimicking the numerical distance effect. Further analysis of the two-way interaction effect between *response hand* × *digit magnitude* revealed a horizontal SNARC effect (see **Figure 7**): in case of relatively small digits (less than 5), MHCT occurred earlier for left hand responses (536 ms) as compared to right hand responses [587 ms; *t*(24) = 3.53, *p* < 0.01]. Vice versa, MHCT in case of relatively large digits (greater than 5) occurred earlier for right hand responses (540 ms) than for left hand responses [584 ms; *t*(24) = 3.34, *p* < 0.01]. *Post hoc* analysis of the *spatial displacement* main effect showed that MHCT occurred earlier for stimuli presented at the border of peripersonal space (554 ms), compared to stimuli presented close to the hands [570 ms; *t*(24) = 3.03, *p* = 0.018]. The comparison with stimuli presented close to the body [564 ms; *t*(24) = 1.56, *p* = 0.266] and in extrapersonal space [560 ms; *t*(24) = 0.79, *p* = 0.439] yielded no significant differences. In general, the pattern of results obtained in MHCT was similar to the observed pattern in RT. Indeed, both measures were highly correlated [*r*(1598) = 0.74, *p* < 0.001]. On average, MHCT occurred only shortly before the actual response [*M_MHCT-RT_* = -29 ms, *SD_MHCT-RT_* = 32 ms; *t*(24) = 4.50, *p* < 0.01].

An analysis of the regression coefficients obtained from the MHCTs yielded a significant main effect of *spatial displacement* [*F*(3,69) = 4.48, *p* = 0.006, $\eta_{p}^{2}$ = 0.16]. The slopes for stimuli presented close to the hands were more inclined than slopes in the border-condition [*t*(24) = -3.47, *p* = 0.024] and in the close-to-body condition [*t*(24) = -3.01, *p* = 0.030]. Again, these results dovetail with the RT pattern (cf. **Table 1**). There were no effects of the group variable *experiment* (*p*s > 0.31).

### IPQ Data

Self-reported ratings of presence (IPQ questionnaire) obtained from the 32 participants were compared with reference data provided by the igroup consortium (see **Table 2**^4^). The reference data set was obtained from video games where the players were equipped with an HMD and comprised 24 mean values for the three subscales. Independent sample *t*-tests yielded a significant difference for spatial presence [*t*(31.31) = 2.08, *p* = 0.022]. Compared to the reference data, participants in our setup reported a higher degree of spatial presence. With respect to involvement and realism, our data compares to the reference (*p*s > 0.206). Together, the results show a sufficient degree of immersion. Improvements with respect to spatial presence dovetail with our earlier results obtained in setups were we applied the LeapMotion^©^ sensor together with an Oculus Rift^©^ DK2 HMD (Schroeder et al., 2016; Lohmann and Butz, 2017).

**Table 2:** Self-report ratings of presence (IPQ questionnaire).

	Observed data		Reference data	
IPQ presence component	*M*	SEM	*M*	SEM
Spatial presence	4.18	0.13	3.46	0.32
Involvement	2.51	0.20	2.59	0.23
Realism	2.30	0.14	2.06	0.26
Mean score	2.99	0.13	2.70	0.17

*Ratings range on seven-point Likert scale from -3 (not at all) to +3 (very much). For the analysis, the value range was recoded to fit a scale from 0 to 6, in accordance with the evaluation guidelines proposed by the igroup consortium.*

To evaluate correlations between the horizontal SNARC effects at different spatial displacements with the subjective presence experience in the virtual environment, Pearson correlation coefficients were computed for each IPQ subscale. Relatively high coefficients were obtained for the spatial presence subscale at all four spatial displacements [*r*(31) = 0.17 to *r*(31) = 0.33]. For stimuli presented close to the hands, the correlation was most pronounced [*r*(31) = 0.331, *p* = 0.064]. The correlations between SNARC effect (as obtained in the regression coefficient analysis) and involvement (|r(31)| < 0.12) and realism [*r*(31) < 0.19] were less pronounced.

## Discussion

In two experiments, we investigated effects of radial distance on numerical magnitude comparisons in an immersive VR. Results show a consistent, but complex pattern of interactions between spatial displacements, numerical magnitude, and side of responding: a horizontal SNARCs effect was observed in terms of faster left-hand responses to relatively small digits and faster right-hand responses for relatively large digits. In kinematic parameters, we also observed the horizontal SNARC effect in terms of response activation in the incorrect hand (MHC) particularly for incongruent trials, shortly before the actual response (MHCT). Regarding the regression analyses, horizontal SNARC effects were most pronounced when target digits were presented close to the hands or in extrapersonal space, compared to other spatial displacements (close to the body or at the border of peripersonal space). Together, these results are in line with the assumption of a situated, sensorimotor representation underlying spatial-numerical associations that supports flexible spatial-numerical mappings.

### The Relationship Between Reachability and SNARC Effects

The results show a robust horizontal SNARC effect for all tested spatial displacements. However, the pattern of results is inconsistent with a linear relationship between numerical magnitude, response side, and physical distance. Instead, regression coefficient analyses revealed that SNARC slopes were most inclined when stimuli were presented near the hands or just outside reachable space. The pronounced SNARC slopes near the hands seem not to be due to a mere near hand effect (Reed et al., 2006), which would predict faster RTs for stimuli presented near the hand in general. Instead, the steeper slopes in this spatial displacement are actually in line with the notion that spatial attention is more specifically subject to altered cognitive processing when objects approach the own hands (Abrams et al., 2008; Tseng et al., 2012). For example, it has been shown that the processing of stimuli close to the hands involves both costs, like delayed disengagement, and benefits, for instance reduced distraction by task-irrelevant features (Davoli and Brockmole, 2012; Liepelt and Fischer, 2016).

In the extrapersonal condition, horizontal spatial-numerical associations were present. This observation may be considered to be in conflict with studies showing that object affordances are limited to peri-personal space (e.g., Costantini et al., 2011; Kalénine et al., 2016). For instance, counting direction preference was reduced when children interacted with counting objects in extrapersonal space using a laser pointer (Patro et al., 2015). However, it is important to emphasize that number presentation within or outside of peripersonal space was task-irrelevant in our study and participants did not perform grasp movements, but classified the presented numbers as being small or large by adjacent left-hand or right-hand closure without further movement, functionally rendering object affordances meaningless for correct responding. Given that Andres et al. (2004) observed an association between grip closure with small numbers, it is still conceivable that using hand closure as response mode in the present experiments induced overall biases in favor of small numbers (and perhaps left space).

So far, there have been no studies that investigated the effects and interactions of different spatial directional codes in the visual presentation dimension on the SNARC effect. If magnitudes in different modalities are mapped directly, one would expect a linear relation between spatial magnitude, e.g., radial distance, and numerical magnitude, which would yield a radial SNARC effect. Our results provide no evidence for such an effect, extending the findings of two earlier studies. Santens and Gevers (2008) had their participants respond to large or small digits with either close or far movements. Close responses were faster for small digits, whereas far responses were relatively faster for large digits. Marghetis and Youngstrom (2014) found evidence for a radial SNARC effect when they let participants respond to positive and negative integers by stepping forward or backward. Here, forward movements yielded faster RTs in case of positive integers, while responses for negative integers were faster in case of backward movements. This compatibility effect vanished when only positive integers were used as stimuli. In both studies, the spatial displacement of the target digits was not manipulated. The results show a semantic overlap between numerical magnitude and response distance (Santens and Gevers, 2008) or between positive- and negative- numbers and response direction (Marghetis and Youngstrom, 2014), respectively. Hence, both results do not necessarly imply a relation between radial distance and SNARC magnitude, but between movement magnitude and SNARC magnitude, that is, a number-action instead of a number-space link. The assumed dominance of a number-action link would also provide an explanation why the effect size of the interactions between horizontal response dimension and the semantic magnitude, which were both task-relevant, are much larger when compared to any effect of the perceptual magnitude in the radial dimension, which was task-irrelevant in both experiments we reported here. Although it was long assumed that numerical magnitude biases cognitive processing automatically, some previous results actually show that very basic perceptual decision tasks can tremendously diminish the influence of spatial-numerical processing (Fias et al., 2001; Schroeder et al., 2017). Moreover, associations between numerical magnitude and radial distance were observed in tasks that positioned effectors accordingly along the distance dimension (Müller and Schwarz, 2007; Gronau et al., 2017).

Theoretically, these results also further specify the taxonomy of spatial-numerical associations, which pits the implicit directional effects as observed in SNARC tasks against other explicit linkages and non-directional links between space and number (Patro et al., 2014; Cipora et al., 2015). We propose that the exact spatial direction of number mappings is determined by situated and task-relevant implementation of action, i.e., using left-hand and right-hand responding, rather than low-level processing of irrelevant spatial information. In the virtual environments, visual cues of depth information (e.g., in terms of number symbol size) further emphasized this type of magnitude information, as closer numbers were larger. However, even this salient relation between numerical magnitude, number symbol size, and distance did not yield interactions reflecting a direct mapping between these types of magnitude information^5^. VR allows to disentangle contributions of these different dimensions and future studies can systematically test this prediction of different situated conditions, which contrasts with previous accounts of generally weaker or steeper vertical SNARC effects. In line with earlier findings (e.g., Andres et al., 2004; Lindemann et al., 2007), these results imply a relationship between action parameters and numerical cognition, which indicates that spatial-numerical associations are realized within a sensorimotor metric. This interpretation is further corroborated by the observed correlation between kinematic parameters (MHC) and numerical magnitude.

### Response Conflict and Effects on Kinematic Parameters

Different experiments have provided evidence for the assumption that the SNARC effect arises at a late, response-related stage of processing (e.g., Keus et al., 2005). For instance, robust SNARC effects have been observed in response-locked event-related potentials (ERPs), while they were absent at earlier ERPs, associated with stimulus processing (Fischer and Miller, 2008). Furthermore, Vierck and Kiesel (2010) showed a compatibility effect between response force and numerical magnitude. Participants responded faster when small digits required a weak response force – while for large digits the opposite was true. Complementary to these findings, our mean hand closure measurements show relatively consistent activations of the incorrect, but SNARC-compatible responses in case of SNARC-incompatible response mappings. Specifically, if participants had to respond to large digits with their left hand, they clenched their right hand significantly stronger than when they had to respond to small digits with their left hand, and vice versa. The temporal pattern of this clenching was highly similar to the RT pattern and showed a similar modulation by spatial displacement. Apparently, response selection was primed by numerical magnitude (see also Daar and Pratt, 2008). Even considering the large interindividual difference in the extent of the SNARC effect (e.g., Wood et al., 2008) and also the amount of response preparation in incorrect hands, which was not reliably available for analysis in one quarter of our participants and also relatively weak (the maximum observed value was ∼0.20, but the threshold for responding was 0.75; see **Figure 7**), this pattern of results again implies a strong grounding of spatial-numerical mappings in a sensorimotor metric. The SNARC slopes for the hand closure did not change with physical distance, however, slopes for the hand closure time showed the same systematicies as slopes obtained from RTs. Given the small sample size, interindividual differences, and the resistance against involuntary hand movements in a forth of our population, it remains open whether response execution was unaffected by reachability. Furthermore, more extensive responses may be better suited to yield variable measures and to detect more subtle interaction terms in future research, as it was also recommended in mouse tracking research (Fischer and Hartmann, 2014; Pinheiro-Chagas et al., 2017).

Regarding the size of effects of numerical magnitude on kinematic parameters, we only observed an effect on the horizontal SNARC effect and apparently the measurement as well as assessment in the VR parameter space was a little noisier, at least as compared to RT assessments. This might indicate a certain specificity of the response parameters. The applied VR setup allowed a convenient manipulation of perceived physical distance and perceived reachability of the target stimuli. Furthermore, motion capture allowed to record a continuous response and to detect SNARC effects within the kinematic parameters of response execution. In general, VR setups seem well-suited to further investigate the role of sensorimotor codes in numerical cognition – especially with respect to different spatial mappings of stimuli and effectors. In future studies, the establishment of the VR procedure and validation for horizontal SNARC effects in the current study allows for further perceptual as well as bodily manipulations, such as the manipulation of the perceived reachability by manipulating the virtual arm length or by the induction of multisensory conflict (e.g., Lohmann and Butz, 2017). As it was argued by Viarouge et al. (2014) spatial-numeric mappings can be established on the fly within different frames of reference, depending on the experimental context, like instructions or saliency of spatial anchors. This impact of situated influences on spatial-numerical mappings led to the formulation of a taxonomy to structure these influences (Cipora et al., 2016). The outlined VR paradigms and manipulations of sensorimotor mappings as well as spatial perception will allow a more detailed investigation of the contextual parameters that give rise to certain spatial-numerical mappings, and to clarify the effect of action-related manipulations on spatial-numerical mappings. For instance, the present results imply that SNARC effects are bound to the response relevant spatial axis, instead of a general dominance of either the horizontal, or the vertical axes.

## Conclusion

Although interactions between semantic and perceptual magnitudes are well-known (e.g., Henik and Tzelgov, 1982; Cohen-Kadosh et al., 2008), the exact shape of these interactions is not clear and corresponding theories were often underspecified, i.e., by generalizing the common code to all possible dimensions. Our results imply that spatial-numeric mappings between different magnitude codes are constrained by task-relevance and characteristics of the sensorimotor metric in which they are realized.

We did not observe interactions between task-relevant horizontal responses and task-irrelevant radial spatial displacements. However, the standard horizontal SNARC effect between task-relevant horizontal responses and task-relevant semantic magnitude was convincingly demonstrated in the immersive VR and further transferred in hand closure measurements to show response competition in the non-responding hand. The systematic manipulation of spatial displacements in the stereoscopic display furthermore revealed a non-linear interaction between physical distance and SNARC magnitude. Given these findings, it seems highly likely that spatial-numerical associations are driven by a sensorimotor metric, which is situated on the fly in the current task-demands. The selective emphasis of action-relevant processing close to effectors is generally consistent with both, theories of anticipatory behavior control and with the parietal foundations of action-relevant numerical processing. The apparent complex interactions, however – particularly when presentations exceeded the peripersonal perceptual space – call for further systematic explorations and theoretical considerations of body-related cognitive processing. Furthermore, the observed tendency for a relation between spatial presence and magnitude of the SNARC effect requires further investigation.

## Ethics Statement

All participants volunteered and provided written informed consent. The study was conducted in accordance with German Psychological Society (DGPs) ethical guidelines (2004, CIII), which are in accordance with the WMA declaration of Helsinki.

## Author Contributions

JL, PS, H-CN, CP, and MB developed the study concept. PS and JL designed the study, performed the data analysis and interpretation, and drafted the manuscript. JL implemented the study, performed the testing, and data collection. H-CN, CP, and MB provided critical revisions. All authors approved the final version of the manuscript for submission.

## Conflict of Interest Statement

The authors declare that the research was conducted in the absence of any commercial or financial relationships that could be construed as a potential conflict of interest.

## Footnotes

1 Each cell mean was obtained from four data points, hence nearly no data points were excluded. The small number of repetitions per condition was the main motivation to rely on median RTs, as the median provides a less biased estimate in case of few observations.

2 This value equals 0 if the hand is open, a value of 1 indicates a clenched fist. The programming interface of the LeapMotion^©^ sensor allows to record this measure directly.

3 Unfortunately, reduced and unequal sample sizes regarding the between factor experiment (N _First_ = 13, N _Second_ = 12) resulted from this procedure. The applied type III sums of square provide an adequate adjustment, but results may still be relatively insensitive regarding possible smaller group differences.

4 http://www.igroup.org/pq/ipq/data.php

5 The apparent absence of effects of physical stimulus size fits well with the results of Longo and Lourenco (2010), who showed that the observed effects of perceptual distance on line-bisection and number-bisection were not affected by changes in physical stimulus size.
